# Sample size calculation in multi-centre clinical trials

**DOI:** 10.1186/s12874-018-0602-y

**Published:** 2018-11-29

**Authors:** Markus Harden, Tim Friede

**Affiliations:** Department of Medical Statistics, University Medical Centre Göttingen, Humboldtallee 32, Göttingen, 37073 Germany

**Keywords:** Block randomisation, Linear mixed model, Random effects

## Abstract

**Background:**

Multi-centre randomized controlled clinical trials play an important role in modern evidence-based medicine. Advantages of collecting data from more than one site are numerous, including accelerated recruitment and increased generalisability of results. Mixed models can be applied to account for potential clustering in the data, in particular when many small centres contribute patients to the study. Previously proposed methods on sample size calculation for mixed models only considered balanced treatment allocations which is an unlikely outcome in practice if block randomisation with reasonable choices of block length is used.

**Methods:**

We propose a sample size determination procedure for multi-centre trials comparing two treatment groups for a continuous outcome, modelling centre differences using random effects and allowing for arbitrary sample sizes. It is assumed that block randomisation with fixed block length is used at each study site for subject allocation. Simulations are used to assess operation characteristics such as power of the sample size approach. The proposed method is illustrated by an example in disease management systems.

**Results:**

A sample size formula as well as a lower and upper boundary for the required overall sample size are given. We demonstrate the superiority of the new sample size formula over the conventional approach of ignoring the multi-centre structure and show the influence of parameters such as block length or centre heterogeneity. The application of the procedure on the example data shows that large blocks require larger sample sizes, if centre heterogeneity is present.

**Conclusion:**

Unbalanced treatment allocation can result in substantial power loss when centre heterogeneity is present but not considered at the planning stage. When only few patients by centre will be recruited, one has to weigh the risk of imbalance between treatment groups due to large blocks and the risk of unblinding due to small blocks. The proposed approach should be considered when planning multi-centre trials.

**Electronic supplementary material:**

The online version of this article (10.1186/s12874-018-0602-y) contains supplementary material, which is available to authorized users.

## Background

When planning a randomized controlled clinical trial, sample size considerations are necessary to assess how many subjects are needed, e.g. to demonstrate a beneficial effect of a new treatment. These considerations usually are based on initial assumptions regarding a clinically meaningful treatment effect, the variability in the data and prespecified type I and type II error rates. Patients are often recruited in more than one centre, for example to account for a low incidence of the disease [1]. Since these centres might differ in some ways, between-centre heterogeneity at baseline needs to be accounted for in the analysis and therefore sample size planning.

When analysing continuous outcomes, baseline differences between centres can be accounted for using either a linear fixed-effects or a linear mixed model. Due to the central limit theorem, sample size calculation can often be based on the normal approximation

1 $$\begin{array}{*{20}l} N=& \frac{\sigma^{2}(k+1)^{2}}{k}\left(\frac{q_{1-\alpha/2}+q_{1-\beta}}{\mu^{*}}\right)^{2} \end{array} $$

where *N* denotes the total sample size, *μ*^∗^ the assumed treatment effect, *σ*^2^ the variance of the observations, *k* the allocation ratio between treatment groups and *q*_*γ*_ the *γ*-quantile of the standard normal distribution [2].

There have been various attempts to extend Formula (1) to multi-centre trials, e.g. by including a multiplicative factor to account for deviations from the standard design. Gallo as well as Ruvuna suggested such an inefficiency factor to account for centre size imbalances in the fixed effects setting [3, 4]. Both methods rely on the proportion of the treatment effects’ variances between balanced and imbalanced centre sizes, where the balanced case gives optimal power.

Van Breukelen and colleagues introduced an inefficiency factor for the mixed model [5]. This factor is based on the relative efficiency of unequal versus equal cluster sizes for the weighted least squares estimator, assuming a linear mixed effects model with an interaction between study site and treatment effect. Fedorov and Jones consider sample size formulas for balanced multi-centre designs and suggest simulations for more complex situations [6]. Vierron and Giradeau suggested a *design-effect* to adjust Eq. (1) for different study designs [7, 8].

All of the approaches mentioned above assume balanced treatment allocation by centre. Randomisation techniques such as block randomisation do not guarantee equal group sizes in all centres, especially if centres are small and block lengths are large. The normal approximation in (1) gives a lower boundary of the necessary sample size, but underpowered trials could occur, especially when between centre heterogeneity is large. We believe that this assumption is too strict for real trials and therefore suggest a sample size formula that accounts for unequal sample sizes.

It has been demonstrated that mixed models tend to yield better results compared to fixed effects models, especially when the number of patients per centre is small [9, 10]. For a small number of centres, however, the fixed effects design might result in better results, because the between-centre variation is likely to be estimated with bias in mixed models in that situation. We therefore aim to construct a sample size formula that accounts for baseline heterogeneity between study centres, assuming a linear mixed model for multi-centre designs.

## Methods

### Statistical model and estimators

We assume a linear mixed-effects model with a fixed intercept *μ*_0_, random effects *u*_*j*_, *j*=1,…,*c* to account for centre heterogeneity at baseline, and a fixed treatment effect *μ*. The data are assumed to follow some continuous distribution allowing for unequal sample sizes. The statistical model is given by

2 $$\begin{array}{*{20}l} Y_{ijk}=&\mu_{0}+u_{j}+\mu\cdot x_{i}+\epsilon_{ijk} \end{array} $$

for pairwise independent *u*_*j*_, *ε*_*ijk*_ with *E*(*u*_*j*_)=0, *Var*(*u*_*j*_)=*τ*^2^<*∞*, *E*(*ε*_*ijk*_)=0, *Var*(*ε*_*ijk*_)=*σ*^2^<*∞*, treatment indicator *x*_*i*_=1_{*i*=2}_ for treatment groups *i*=1,2, centres *j*=1,…,*c* and individuals *k*=1,…,*n*_*ij*_ for each treatment-centre combination. The shared random effect *u*_*j*_ within centres induces the following covariance matrix $Cov(Y_{111}, \ldots, Y_{2cn_{2c}})=\bigoplus _{j=1}^{c}\left [\sigma ^{2}\mathbf {I}_{n_{j}}+\tau ^{2}\mathbf {J}_{n_{j}}\right ]$, including all *N* observations with $N=\sum _{i=1}^{2}\sum _{j=1}^{c}n_{ij}$. Here, $\mathbf {I}_{n_{j}}$ denotes the *n*_*j*_-dimensional identity matrix and $\mathbf {J}_{n_{j}}$ the *n*_*j*_-dimensional matrix consisting of ones only with *n*_*j*_=*n*_1*j*_+*n*_2*j*_. We assume zero risk of contamination of the control group.

We are interested in differences between treatment groups and test the null hypothesis *H*_0_:*μ*=0 against the two-sided alternative *H*_*A*_:*μ*≠0. The distribution of the estimated treatment effect $\widehat {\mu }$ can be approximated by a normal distribution, if the sample size is sufficiently large (say sample sizes larger 30). It follows

3 $$\begin{array}{*{20}l} T=&\frac{\widehat{\mu}}{\sqrt{\widehat{\text{Var}\left(\widehat{\mu}\right)}}}\stackrel{H_{0}}{\sim} \mathrm{N}(0,1). \end{array} $$

The null hypothesis can be rejected if the test statistic |*T*| exceeds the quantile *q*_1−*α*/2_ of the reference distribution for some fixed type I error rate *α*∈(0,1). In order to apply the statistical test, suitable estimators for the unknown parameters have to be chosen.

We choose $\widehat {\mu }=\overline {Y}_{2\cdot \cdot }-\overline {Y}_{1\cdot \cdot }$ to measure treatment group differences, where $\overline {Y}_{i\cdot \cdot }=\frac {1}{N_{i}}\sum _{j=1}^{c}\sum _{k=1}^{n_{ij}}Y_{ijk}$ denotes the group mean in treatment group *i*. This estimator is unbiased, even if centres recruited patients for one treatment group only. The variance of ${\widehat {\mu }}$ can be written as

4 $$\begin{array}{*{20}l} \text{Var}\left(\widehat{\mu}\right)=&\sigma^{2}\frac{N}{N_{1}N_{2}}+\tau^{2}\sum_{j=1}^{c}\left(\frac{n_{1j}}{N_{1}}-\frac{n_{2j}}{N_{2}}\right)^{2}. \end{array} $$

Details on the derivation can be found elsewhere [8].

$\text {Var}\left (\widehat {\mu }\right)$ depends on the overall sample size *N*, the variances *σ*^2^ and *τ*^2^, and additionally the sample sizes by treatment group (*N*_1_, *N*_2_), number of study centres (*c*), and the sample sizes within study centres (*n*_1*j*_,*n*_2*j*_). In case of a perfectly balanced randomisation, the differences between centres cancel out and the treatment effect’s variance only depends on sample sizes *N*, *N*_1_, *N*_2_ and variance *σ*^2^, resulting in a sample size formula similar to (1).

The unknown variance parameters *τ*^2^ and *σ*^2^ can be estimated using the following quadratic forms

$$\begin{array}{*{20}l} \widehat{\sigma}^{2}=&\frac{1}{2 c}\sum_{i=1}^{2}\sum_{j=1}^{c}\frac{1}{n_{ij}-1}\sum_{k=1}^{n_{ij}}\left(Y_{ijk}-\overline{Y}_{ij\cdot}\right)^{2}\\ \widehat{\tau}^{2}=&\frac{1}{2}\sum_{i=1}^{2}\frac{1}{c-1}\sum_{j=1}^{c}\left(\overline{Y}_{ij\cdot}-\overline{Y}_{i\cdot\cdot}\right)^{2}. \end{array} $$

### Additional assumptions for sample size calculation

*N*_*i*_ and *n*_*ij*_ are determined by recruitment and treatment allocation. We aim to replace all *N*_*i*_, *n*_*ij*_ in (4) by their expectations that can be calculated based on the randomisation procedure and planned allocation proportion between treatment groups.

In the following, we want to calculate the overall sample size *N* and assume

1. a block randomisation stratified by centre with fixed block length *b*,
2. *k*:1 allocation ratio at each study site for *k*∈IN,
3. proportion of overall sample sizes between treatment groups according to allocation: *N*_1_=*kN*_2_.

### Block randomisation

Since randomisation will not always result exactly in the planned allocation, we take a closer look at the randomisation process. Block randomisation with fixed block length *b* is a procedure where every *b* subjects get randomised between treatment groups at a time [11]. Complete blocks do always fulfil the planned *k*:1 allocation ratio. The block size should be unknown to investigators to strengthen the blinding in the trial.

In this article, we assume patients to be assigned to treatment groups *i*=1,2 within centres, for a fixed *k*:1 allocation ratio. This means that in each randomisation block *b* patients are randomized between treatment groups 1 and 2 in a way that for each patient receiving treatment 2, *k* patients will receive treatment 1. The set of randomisation tuples $\Pi ^{k}_{b}$ depends on block length *b* and allocation parameter *k*. It is defined as follows

5 $$\begin{array}{*{20}l} \Pi^{k}_{b}=& \left\{{\vphantom{\sum_{\ell=1}^{b}}}(x_{1}, \ldots, x_{b})\in\Pi_{b} \Bigg|\right.\\ & \left.\sum_{\ell=1}^{b}1_{\{x_{\ell}=1\}}=\frac{kb}{k+1}=b-\sum_{\ell=1}^{b}1_{\{x_{\ell}=2\}}\right\} \end{array} $$

where *Π*_*b*_:={(*x*_1_,…,*x*_*b*_)|*x*_*ℓ*_∈{1,2}}.

Treatment allocation imbalances can only occur in incomplete blocks with an upper boundary of *kb*/(*k*+1). The choice of *k* can be based on several assumptions as ethics, costs and other factors and will not be discussed further in this article. This topic is covered in more detail in a review by Dumville and colleagues [12]. It is available in many software packages and is therefore easy to apply [13, 14]. Further advantages and disadvantages of block randomisation are considered in the Discussion.

### Derivation of the sample size formula

The underlying idea of sample size calculation is to find the overall sample size *N*, such that the quantile *q*_1−*α*/2_ of the reference distribution under the null hypothesis equals the quantile $q^{*}_{\beta }$ of the reference distribution under a fixed alternative for type I and II error rates *α* and *β*.

Since we do assume a normally distributed test statistic, $q^{*}_{\beta }$ can be approximated by a shifted N(0,1)-quantile $q^{*}_{\beta }\approx q_{\beta }+\frac {\mu }{\text {Var}(\mu)}$ resulting in the following equation to construct a sample size formula

6 $$\begin{array}{*{20}l} (q_{1-\alpha/2}+q_{1-\beta})^{2}=&\frac{\mu^{2}}{\text{Var}(\mu)}. \end{array} $$

By isolating the sample size *N*, which is part of Var(*μ*), one can derive a sample size formula. In case of an ideal allocation, i. e., *n*_1*j*_=*kn*_2*j*_ for all centres, (6) is equal to (1). Since this is unlikely to be observed, unbalanced designs are taken into account by incorporating expectations with respect to the randomisation procedure.

**Fig. 1:**
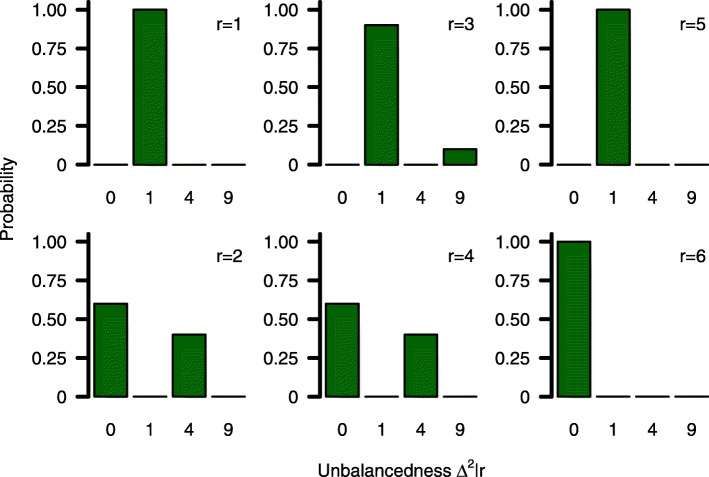
Probability distribution. Conditional probability distributions of *Δ*^2^|*r* for varying numbers of randomized subjects *r*=1,…,*b*=6

We derive the sample size formula for the general case of a *k*:1 allocation ratio and assume the overall treatment group sample sizes to fulfil *N*_1_=*kN*_2_ and therefore *N*=(*k*+1)*N*_2_. This leads to the set of randomisation tuples in (5). By taking assumptions 1-3, the variance of $\widehat {\mu }$ given in (4) simplifies to

7 $$\begin{array}{*{20}l} \text{Var}\left(\widehat{\mu}\right)=&\frac{\sigma^{2}(k+1)^{2}}{k N}+\frac{\tau^{2}(k+1)^{2}}{N^{2}}\sum_{j=1}^{c}\Delta^{2}_{j} \end{array} $$

where $\Delta ^{2}_{j}{:=}\left (\frac {n_{1j}}{k}-n_{2j}\right)^{2}\in \left [0, m^{*}\right ]$ describe each centre’s imbalance that will result from incomplete blocks with *m*^∗^=*b*^2^/(*k*+1)^2^. The (discrete) probability distribution of $\Delta _{j}^{2}$ depends on $\Pi _{b}^{k}$ and the number of patients in the last block which equals the remainder of the Euclidean division *r*_*j*_=*n*_*j*_ mod *b*. An example of $\Delta _{j}^{2}|r_{j}$ for a single centre is illustrated in Fig. 1.

**Fig. 2:**
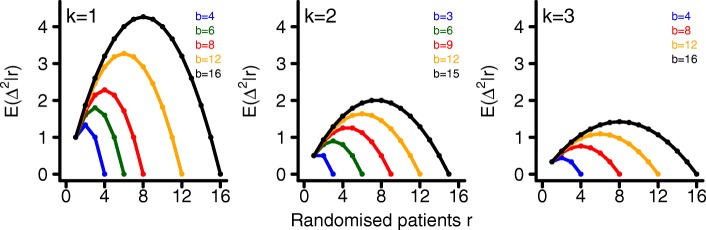
Expectation of *Δ*^2^|*r*. Conditional expected imbalance between treatment groups for allocation parameter *k*=1,2,3 and various numbers of subjects *r*=1…,*b* and block lengths *b*

The probability distribution of $\Delta _{j}^{2}|r_{j}$ is fully described by block length *b*, allocation parameter *k* and *r*_*j*_. For planning purposes it therefore seems reasonable to replace $\Delta _{j}^{2}|r_{j}$ by its expectation $\text {E}\left (\Delta _{j}^{2}|r_{j}\right)$ to eliminate sample sizes *n*_1*j*_ and *n*_2*j*_ from (7). The expectation of the probability distribution can easily be derived as

8 $$\begin{array}{*{20}l} \text{E}\left(\Delta^{2}_{j}|r_{j}\right)=&\frac{1}{m^{*}}\sum_{\ell=0}^{m^{*}}p(\ell|r_{j})\cdot \ell \end{array} $$

where *p*(·|*r*_*j*_) denotes the conditional density function of $\Delta _{j}^{2}|r_{j}$. These expectations are shown in Fig. 2 for a single randomization block, *k*=1,2,3 and various block lengths *b*. Since the expected imbalance is the largest for *k*=1 we will restrict simulations to this case.

The expected imbalance between treatment groups $\text {E}\left (\Delta ^{2}_{j}|r_{j}\right)$ increases with block length. This happens due to the fact that the probability to receive an incomplete randomisation block increases with increasing block length *b*. It is maximised when the last randomisation block only consists of $\frac {b k}{k+1}$ patients receiving treatment 1 or $\frac {b}{k+1}$ patients receiving treatment 2, respectively. If we replace $\Delta _{j}^{2}|r_{j}$ by $\text {E}\left (\Delta _{j}^{2}|r_{j}\right)$, we can transform (6) into the following sample size formula for multi-centre trials (derivation is given in the appendix, see Additional file 1)

9 $$\begin{array}{*{20}l} &N^{k}_{\text{MC}}=\frac{\sigma^{2}(k+1)^{2}}{2k}\left(\frac{q_{1-\alpha /2}+q_{1-\beta}}{\mu}\right)^{2}\\ &+\sqrt{\frac{\sigma^{4}(k+1)^{4}}{4 k^{2}} + \frac{\tau^{2}(k+1)^{2}\mu^{2} \sum_{j=1}^{c}\ \text{E}\left(\Delta_{j}^{2}|r_{j}\right)}{\left(q_{1-\alpha /2}+q_{1-\beta}\right)^{2}}}\\ &\cdot\left(\frac{q_{1-\alpha /2}+q_{1-\beta}}{\mu}\right)^{2}.\notag \end{array} $$

### Simulations

#### General settings

We perform a simulation study to assess the accuracy of the sample size formulas in terms of statistical power using R, version 3.3.1 [15]. For each scenario, we repeat *n*_sim_=10,000 independent simulation runs. The R package blockrand is used for block randomisation [13]. All data are generated based on the statistical model described in (2), assuming a normal distribution for *u*_*j*_ and *ε*_*ijk*_. The test statistic *T* given in (3) is used for all power simulations and it is approximated by a standard normal distribution under the null hypothesis. The effect of block randomisation is strongest for *k*=1, we therefore present simulation results for this setting only. The assumed effect size *μ*^∗^ and values for variance components *σ*^2^ and *τ*^2^ are based on an example trial described in the next section. All code used for simulations is available in the Appendix, see Additional files 2, 3, 4, 5, 6, 7 and 8.

#### Subject-to-centre allocation

We consider equally as well as unequally sized study centres based on the following methods.

1. *Equally sized study centres:* Only in this situation, we can predict the sample size very precisely, since we can specify *r*_*j*_ correctly prior to recruitment. Here, study centres are assumed to be equal. Since this assumption is limited to the fixed overall sample size *N*, we distribute the overall sample size to centres as follows
  $$\begin{array}{*{20}l} \text{Equal:}\ n_{j}=&\left\lfloor\frac{N}{c}\right\rfloor+1_{\{j\leq m\}} \end{array} $$
  for *m*=*N* mod *c* and *j*=1,…,*c*.
2. *Unequally sized study centres:* In most experiments, the true allocation of patients cannot be foreseen. Recruitment rates can be estimated beforehand, but since only the number of patients in the last randomisation block affects the presented sample size formulas, no precise estimation of unbalanced treatment allocation can be made. To model this situation, study centres are assumed to be unequal but limited to the fixed overall sample size *N*. We use a multinomial distribution to generate unequal sample sizes by centre, assuming the following scenarios

$$\begin{array}{*{20}l}\text{Unequal 1: }&\left(n_{1}, \ldots, n_{c}\right)'\sim\ \text{Multi}_{c}\left(N, p_{1}, \ldots, p_{c}\right)\\ &\text{with }p_{j}=\frac{1}{c}\\ \text{Unequal 2: }&\left(n_{1}, \ldots, n_{c}\right)'\sim\ \text{Multi}_{c}\left(N, p^{*}_{1}, \ldots, p^{*}_{c}\right)\\ &\text{with }p^{*}_{j}=\frac{p_{j}}{\sum_{k=1}^{c}p_{k}}\ \text{and}\ p_{j}\stackrel{iid}{\sim} U[0; 1]. \end{array} $$

### Example: The COMPETE II trial

Multi-centre trials are applied in many different disease areas. We present an example in the setting of disease management systems and use this trial to illustrate the sample size approach proposed.

Holbrook and colleagues conducted a randomized, multi-centre trial to investigate the benefit of an individualized electronic decision support system in adult patients diagnosed with type 2 diabetes [16]. This new intervention provided patient specific summaries and recommendations based on electronic medical records, aiming to improve the quality of diabetes management between patients and general practitioners. The tool was integrated into the practice work flow and offered web-based access by patients. In addition, an automated telephone reminder system was provided and all patients received a colour-coded printout quarterly. The control treatment consisted of usual care without use of this tool.

Primary outcome was a composite score difference compared to baseline. The composite score measured process quality on a scale from 0 to 10, based on the following parameters: blood pressure, cholesterol, glycated haemoglobin, foot check, kidney function, weight, physical activity, and smoking behaviour. The clinical targets are described in the original article. It was assessed at baseline and 6 months after randomization.

For this trial, 511 patients from 46 primary care providers were randomly assigned to intervention or control. At planning stage, the investigators aimed to recruit 508 patients to achieve 80% power to detect a difference of 1 for the primary outcome between treatment groups using a two-sided t-test with a type-1 error rate of *α*=0.05. No information on the assumed standard deviation is given in the article. Block-randomisation was stratified by study site in blocks of six, following a 1:1 allocation scheme.

The absolute measured improvement of composite scores between treatment groups was 1.26 (95% confidence interval (CI) 0.79-1.75; *p*-value < 0.001) favouring the new intervention.

## Results

### Approaches to sample size calculation

As long as no values for *r*_*j*_ are assumed, Formula (9) cannot be used for sample size calculation. We consider a setting, where each centre will have at most one incomplete randomisation block. In the following, we present different ways to specify values of $\text {E}\left (\Delta _{j}^{2}|r_{j}\right)$ for each centre prior to recruitment.

The resulting sample size formulas are listed in Table 1. More detailed explanations are provided in the following subsections.

**Table 1:** Overview of sample size formulas

Lower boundary	$N^{k}_{\text {lower}}$ = $\left (\frac {q_{1-\alpha /2}+q_{1-\beta }}{\mu }\right)^{2}\left (\frac {\sigma ^{2}(k+1)^{2}}{k}\right)$
Equal centres	$N^{k}_{\mathrm {MC,E}}$ = $\left (\frac {q_{1-\alpha /2}+q_{1-\beta }}{\mu }\right)^{2}\left (\frac {\sigma ^{2}(k+1)^{2}}{2k}+\sqrt {\frac {\sigma ^{4}(k+1)^{4}}{4 k^{2}} + \frac {\tau ^{2}(k+1)^{2}\mu ^{2} c\ \text {E}\left (\Delta _{1}^{2}|r_{1}\right)}{\left (q_{1-\alpha /2}+q_{1-\beta }\right)^{2}}}\,\right)$
Unequal centres	$N^{k}_{\mathrm {MC,U}}$= $\left (\frac {q_{1-\alpha /2}+q_{1-\beta }}{\mu }\right)^{2}\left (\frac {\sigma ^{2}(k+1)^{2}}{2k}+\sqrt {\frac {\sigma ^{4}(k+1)^{4}}{4 k^{2}} + \frac {\tau ^{2}(k+1)^{2}\mu ^{2} c\overline {\text {E}\left (\Delta _{1}^{2}|\cdot \right)}}{\left (q_{1-\alpha /2}+q_{1-\beta }\right)^{2}}}\,\right)$
Upper boundary	$N^{k}_{\text {upper}}$ = $\left (\frac {q_{1-\alpha /2}+q_{1-\beta }}{\mu }\right)^{2}\left (\frac {\sigma ^{2}(k+1)^{2}}{2k}+\sqrt {\frac {\sigma ^{4}(k+1)^{4}}{4 k^{2}} + \frac {\tau ^{2}(k+1)^{2}\mu ^{2} c\ \text {E}\left (\Delta _{1}^{2}\middle |\frac {b}{k+1}\right)}{\left (q_{1-\alpha /2}+q_{1-\beta }\right)^{2}}}\,\right)$

Lower and upper boundaries as well as sample size formulas for equal and unequal centre sizes

#### Lower boundary

Given an ideal treatment allocation (*n*_1*j*_=*kn*_2*j*_ for all centres), potential centre differences cancel out and do not affect the statistical power of the trial. In this case, sample size formula (1) would be suitable to plan the trial. However, the trial still had to be analysed with a mixed effects model to get an unbiased estimate for Var(*μ*), since Var(*Y*_*ijk*_)=*σ*^2^+*τ*^2^. This formula is the standard approach to sample size calculation and is often used to plan multicentre trials. It therefore serves as one reference for sample size calculation and power simulations.

#### Equal centre sizes

The exact number of patients by study centre will be unknown at the planning stage, but in some situations, it might be reasonable to assume centres to be equally large. In this scenario, the sum of expected differences simplifies to a single quantity

10 $$\begin{array}{*{20}l} \sum_{j=1}^{c}\text{E}\left(\Delta_{j}^{2}|r_{j}\right)\approx c\cdot\text{E}\left(\Delta_{1}^{2}|r_{1}\right) \end{array} $$

which needs to be specified for sample size estimation.

Since the overall sample size *N* and the number of subjects in the last randomisation block depend on each other, *r*_1_ still has to be specified. The unspecified value of $\text {E}\left (\Delta _{1}^{2}|r_{1}\right)$ can be determined by calculating the sample size $N^{k}_{\mathrm {MC,E}}$ for each *r*_1_∈{1,…,*b*}. Keep $N^{k}_{\mathrm {MC,E}}\left (r_{1}^{*}\right)$ with

11 $$\begin{array}{*{20}l} r_{1}^{*}=&\underset{r_{1}}{\text{argmin}} \left|\left(\frac{N^{k}_{\mathrm{MC,E}}(r_{1})}{c} \ \text{mod}\ b\right)-r_{1}\right|. \end{array} $$

#### Unequal centre sizes

For the general case, we suggest using the average of the expected imbalance for each centre

12 $$\begin{array}{*{20}l} \text{E}\left(\Delta_{j}^{2}|r_{j}\right)\approx&\frac{1}{b}\sum_{\ell=1}^{b} \text{E}\left(\Delta_{1}^{2}|\ell\right){=:}\overline{\text{E}\left(\Delta_{1}^{2}|\cdot\right)}. \end{array} $$

This basically assumes a univariate distribution of *r*_*j*_ on [1,…,*b*].

#### Upper boundary

We can identify an upper boundary of the sample size, given that all parameters are specified correctly at planning stage. The maximal imbalance between treatments would occur, if each centre recruited and allocated *r*_*j*_=*b*/(*k*+1) or *r*_*j*_=*kb*/(*k*+1) subjects to a single treatment group resulting in $\Delta _{j}^{2}=m^{*}$. Therefore, the most conservative sample size calculation will be performed with the following approximation

13 $$\begin{array}{*{20}l} \sum_{j=1}^{c}\text{E}\left(\Delta_{j}^{2}|r_{j}\right)\approx c\cdot\text{E}\left(\Delta_{1}^{2}\middle|\frac{b}{k+1}\right). \end{array} $$

### Example: Sample size calculation

To give an example of the application of the proposed sample size formula, we demonstrate the effects of between-centre heterogeneity combined with incomplete block randomisation based on the COMPETE II trial.

**Fig. 3:**
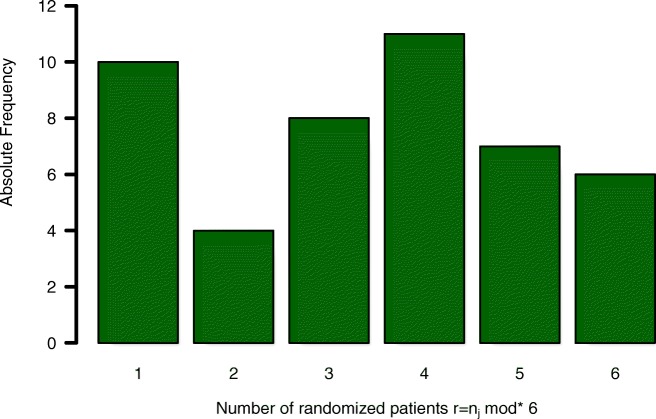
Example: Block randomisation. Number of final randomisation blocks by centre with block length *b*=6 based on the COMPETE II trial [17]

The number of patients per study site is reported in [17]. Based on those numbers, we know the completeness of all randomisation blocks by centre as shown in Fig. 3.

We use these numbers to illustrate the influence of unbalanced treatment group allocation on sample size and statistical power based on the assumed model. In total, 40 incomplete randomisation blocks (*r*<6) out of 46 study sites occurred. Based on the assumed model, statistical power of the analysis might be reduced due to those 40 incomplete randomisation blocks, compared to a trial, where the same amount of patients would have been recruited at a single centre.

**Fig. 4:**
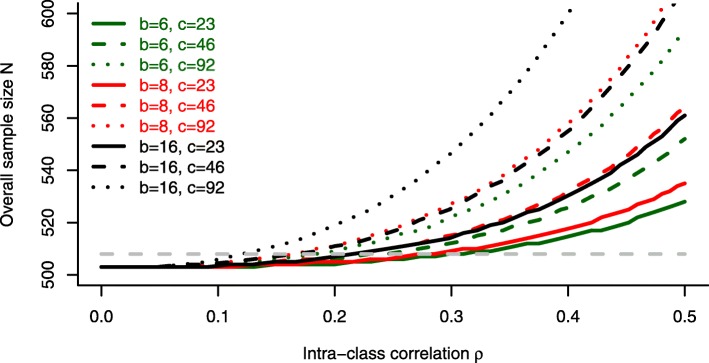
Example: Sample size based on $N_{\mathrm {MC,U}}^{1}$. Derived for *μ*=1, *σ*=4, varying block length *b*, number of centres *c* and intra-class correlation *ρ*. Dashed grey line represents the planned sample size of the trial (*N*=508)

If we were to plan a new trial with similar features (*μ*=1, *σ*=4, type 1 and type 2 error rates *α*=0.05 and *β*=0.2, respectively) we could plug these values into the sample size formula $N_{\mathrm {MC,U}}^{1}$ for unequal centre sizes. The assumption of uniformly distributed values of *r*_*j*_ on [1,…,*b*] could be underpinned by a *χ*^2^-test for goodness-of-fit (*p*=0.4159). The influence of intra-class correlation *ρ*∈[0,0.5], number of centres *c*∈{23,46,92} and block lengths *b*∈{6,8,16} on the sample size is shown in Fig. 4.

For a block length of *b*=6, as chosen in the trial, no substantial influence of the intraclass correlation *ρ* on the overall sample size can be observed. The reported value of *ρ*=0.08 in the trial would not require a sample size adjustment compared to the standard approach ($N^{k}_{\text {lower}}$). For larger block lengths, however, a strong increase of the estimated sample size can be seen, especially for *ρ*>0.2 and an increasing number of centres.

### Power simulations

**Fig. 5:**
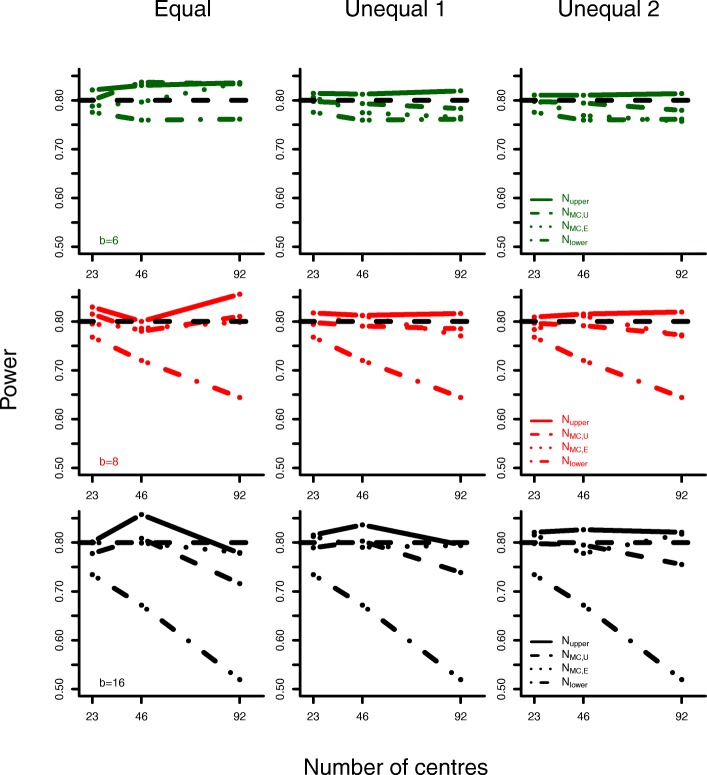
Example: Power simulations. Simulated power based on the planned sample sizes for *μ*=1, *σ*=4, *ρ*=0.5, varying numbers of centres and varying block lengths. Dashed black line represents the targeted power of 0.8

In addition to sample size calculations, we present some power simulation results for parameter settings based on the COMPETE II trial. We specify a treatment effect of *μ*=1, standard deviation *σ*=4 and intraclass-correlation coefficient *ρ*=0.5. Data was generated for various block lengths, numbers of centres and subject-to-centre allocation schemes. Resulting sample sizes and associated statistical power are given in Table 2 and Fig. 5.

**Table 2:** Example: Comparison of sample size formulas

Block length	Formula	Number of centres		
		c=23	c=46	c=92
6	$N^{k}_{\text {lower}}$	503	503	503
	$N^{k}_{\mathrm {MC,E}}$	525	524	569
	$N^{k}_{\mathrm {MC,U}}$	528	552	594
	$N^{k}_{\text {upper}}$	541	575	634
8	$N^{k}_{\text {lower}}$	503	503	503
	$N^{k}_{\mathrm {MC,E}}$	525	587	606
	$N^{k}_{\mathrm {MC,U}}$	535	564	616
	$N^{k}_{\text {upper}}$	551	592	662
16	$N^{k}_{\text {lower}}$	503	503	503
	$N^{k}_{\mathrm {MC,E}}$	586	603	762
	$N^{k}_{\mathrm {MC,U}}$	561	610	692
	$N^{k}_{\text {upper}}$	587	654	762

Derived for *μ*=1,*σ*=4,*ρ*=0.5, varying block lengths and varying numbers of centres

Analyses based on the lower boundary formula do not achieve the planned power of 0.8. The deviance from the nominal level increases with block length and number of centres. The upper boundary formula results in power levels that exceed the nominal level slightly.

The new sample size formulas for equal and unequal centre sizes lead to reasonable power simulation results. Even if subject allocation is performed randomly in each simulation run (Unequal 2), adequate statistical power can be obtained.

Subject allocation might, by chance, result in more complete randomisation blocks for $N_{\mathrm {MC,U}}^{1}$ than $N_{\text {upper}}^{1}$. This leads to some situations where the formula with smaller sample size $\left (N_{\mathrm {MC,U}}^{1}\right)$ achieves higher (estimated) power. This observation underlines the necessity to take block length and patient recruitment into consideration when planning large multi-centre trials.

## Discussion

Patient enrolment and treatment allocation are key elements of every successful clinical trial. Randomisation techniques are used to achieve comparable treatment groups minimizing the risk of selection bias. Unfortunately, these randomisation procedures can result in unequal treatment group sizes and therefore a power loss compared to a balanced trial. Such imbalances cannot be determined prior to the trial, but we presented a way to estimate these values based on expectations.

The ICH E9 Guideline encourages the use of block randomisation and states the following on the choice of block sizes [1]: *”Care should be taken to choose block lengths that are sufficiently short to limit possible imbalance, but that are long enough to avoid predictability towards the end of the sequence in a block. Investigators and other relevant staff should generally be blind to the block length; the use of two or more block lengths, randomly selected for each block, can achieve the same purpose.”*

The results shown remain valid when using variable block sizes, since incomplete blocks can occur using either method. Only the determination of expected values E(*Δ*^2^|*r*) is more complicated for variable blocks, because it depends on the range of block sizes used.

In a recent systematic review on prevalence and reporting of recruitment, randomisation and treatment errors in phase III randomized, controlled trials, stratified block randomisation was identified as randomisation technique of choice in 50% of 82 included studies published in New England Journal of Medicine, Lancet, Journal of the American Medical Association, Annals of Internal Medicine, or British Medical Journal between January and March 2015 [18]. The median number of participants per trial was 650 (range 40-84,496) and the number of centres varied between 1 and 1,161 with a median of 24. There are a number of trials that used fixed block lengths greater than 10 to allocate subjects between two treatment groups [19, 20]. Trials using random block randomisation almost never report the underlying block sizes, therefore we can not compare fixed versus random blocks any further. Overall, these observations support our idea to account for incomplete blocks to plan a multi-centre trial using either method for randomisation.

One limitation of our approach is a lack of knowledge on centre heterogeneity at the planning stage. There have been various articles with estimates of intraclass-correlation coefficients (ICC) derived from cluster-randomized trials. These estimates can be used to get an initial guess for centre heterogeneity in multi-centre trials. A nice overview is given in the following text book [21]. Also, the implementation of an adaptive sample size reestimation procedure could account for this problem as it has been applied for cluster randomized trials and the fixed effects multi-centre trial design [22–24]. The development of sample size reestimation strategies based on the approach proposed here is subject to ongoing research. When planning an individually-randomized multi-centre trial there is a risk of control group contamination. This can partly be handled in placebo-controlled pharmacological trials or when proper blinding of patients and researchers is implemented [25]. Alternatively a cluster-randomized trial could be used to prevent contamination of treatment groups. This would, however, be associated with a higher sample size compared to the multi-centre design [26].

Here, we assumed a constant treatment effect across the centres. This is in line with the ICH E9 Guideline, which demands to avoid treatment-by-centre interactions in the primary analysis [1]. Therefore, this is at least for the planning of a trial an adequate assumption. Nevertheless, sensitivity analyses might explore treatment-by-centre interactions. Extending the sample size approach to a model including treatment-by-centre interactions is subject to future research.

## Conclusion

Imbalances in treatment allocation will lead to a power loss in multi-centre trials, if baseline heterogeneity is present. This risk can be accounted for when using appropriate methods for sample size calculation. To reduce uncertainty of sample size calculation, we recommend to calculate lower and upper boundaries in addition to the sample size.

## Additional files

Additional file 1 This file contains the calculation of the sample size formula. (PDF 24 kb)

Additional file 2 This file contains all source code used for simulations. (R 13 kb)

Additional file 3 This file contains the code used for Fig. 1. (R 3 kb)

Additional file 4 This file contains the code used for Fig. 2. (R 13 kb)

Additional file 5 This file contains the code used for Fig. 3. (R 1 kb)

Additional file 6 This file contains the code used for Fig. 4. (R 4 kb)

Additional file 7 This file contains the code used for Fig. 5. (R 12 kb)

Additional file 8 This file contains the code used for Table 2. (R 1 kb)

## Abbreviations

CI: Confidence interval
ICC: Intraclass-correlation coefficient
