# Supplementary material for: Sample size calculation in multi-centre clinical trials
Source: BMC Med Res Methodol. 2018 Nov 29;18:156. doi: 10.1186/s12874-018-0602-y (PMC6267841; doi:10.1186/s12874-018-0602-y)
Supplement: Supplementary file 1 — This file contains the calculation of the sample size formula. (PDF 24 kb) [file 12874_2018_602_MOESM1_ESM.pdf]

Appendix

Calculation of the sample size formula

When combining (6) and (7) and replacing $\Delta_j^2|r_j$ by it's expectation, the following calculations lead to the overall sample size formula (9).

$$
\begin{aligned}
(q_{1-\alpha/2} + q_{1-\beta})^2 &= \frac{\mu^2}{\mathrm{Var}(\mu)} \\
\Longleftrightarrow N^2 - N\frac{\sigma^2(k+1)^2}{k}&\left(\frac{q_{1-\alpha/2} + q_{1-\beta}}{\mu}\right)^2 \\
&= \tau^2(k+1)^2 \sum_{j=1}^{c} \mathrm{E}\left(\Delta_j^2|r_j\right)\left(\frac{q_{1-\alpha/2} + q_{1-\beta}}{\mu}\right)^2
\end{aligned}
$$

This quadratic equation can be solved with standard algebra techniques and leads to the following result

$$
\begin{aligned}
N_{1,2} &= \frac{\sigma^2(k+1)^2}{2k}\left(\frac{q_{1-\alpha/2} + q_{1-\beta}}{\mu}\right)^2 \\
&\pm\sqrt{\frac{\sigma^4(k+1)^4}{4k^2} + \frac{\tau^2(k+1)^2\mu^2 \sum_{j=1}^{c} \mathrm{E}\left(\Delta_j^2|r_j\right)}{\left(q_{1-\alpha/2} + q_{1-\beta}\right)^2}} \\
&\cdot \left(\frac{q_{1-\alpha/2} + q_{1-\beta}}{\mu}\right)^2 .
\end{aligned}
$$

Since we are interested in positive integers, we take the positive solution from above. □
